# Willingness to Receive Latent Tuberculosis Infection Treatment Among Migrants in Tijuana, Baja California, Mexico

**DOI:** 10.1007/s10903-026-01891-2

**Published:** 2026-03-08

**Authors:** Richard S Garfein, Marva Seifert, Daniela Abramovitz, Ietza Bojorquez-Chapela, Alicia Harvey-Vera, Jaime Sepulveda, Steffanie A Strathdee

**Affiliations:** 1 Herbert Wertheim School of Public Health and Human Longevity Science, University of California, San Diego, San Diego, USA; 2 School of Medicine, University of California, San Diego, San Diego, USA; 3 Department of Population Studies, El Colegio de la Frontera Norte, Tijuana, Mexico; 4 Institute for Global Health Sciences, University of California, San Francisco, San Francisco, USA

**Keywords:** Immigration, Health Knowledge and Attitudes, Substance Use, Tuberculosis Prevention, Homeless Persons

## Abstract

Over 80% of tuberculosis cases in the United States are due to reactivation of untreated latent tuberculosis infection (LTBI), most of whom were foreign born. LTBI treatment can greatly lower the risk of progression to active TB disease, and shorter treatment regimens increase the potential of migrants and other persons in vulnerable conditions, to complete treatment. We conducted across-sectional study to assess willingness to complete a one-month course of LTBI treatment among internal and international migrants living in Tijuana, BC, Mexico, and to identify factors associated with treatment unwillingness between November 2020 and April 2021. Prior to administering TB skin tests (TST), participants were asked if they would accept LTBI treatment if indicated by test results and clinical examination. Recruitment occurred in migrant shelters throughout Tijuana and at a storefront office conducting research and harm reduction services for people who use drugs. Among 595 participants, 80.4% were living in shelters, 3.5% were recruited at shelters but living independently, and 16.1% were recruited through the storefront office. Overall, 71 (11.9%) indicated that they were unwilling to take LTBI treatment and 109 (18.3%) had positive TST results. Unwillingness to take LTBI treatment was more common among participants from the storefront office compared to the shelter (64.6% vs. 1.8%, p<0.001). Since only 1.8% of shelter participants were unwilling to receive treatment, multivariable Poisson regression with robust variance estimation via GEE for identifying factors independently associated with LTBI treatment unwillingness was restricted to storefront participants. Treatment unwillingness among storefront participants was positively associated with perceiving health status as “good” or “very good” (prevalence ratio [PR]=2.37, 95% confidence interval [CI]: 1.52, 3.99) and heroin use in the past six months (PR=1.70; 95% CI: 1.33, 2.19). Unstable housing was reported as a barrier to treatment. These findings suggest that most migrants in Tijuana were willing to receive short-course LTBI treatment if indicated, yet willingness was lower among those at greatest risk of TB. Efforts to increase testing and treatment, as well as further research on overcoming barriers to treatment willingness are needed.

## Background

Tuberculosis (TB) is a major global health concern, causing 10 million cases and nearly one million deaths annually, making it a leading infectious disease killer.^1^ Approximately 5% of *Mycobacterium tuberculosis* infections progress to TB within two years, while the remainder have a 5% lifetime reactivation risk. Those with risk factors like HIV, diabetes, undernutrition, and smoking face higher reactivation risks.^2^ In low incidence countries such as the United States (U.S.), LTBI reactivation is the leading source of new TB cases.^3^ Migrants in the U.S./Mexico border region are particularly vulnerable due to unstable housing, healthcare access barriers, and socio-economic conditions.^4^–^6^.

The U.S./Mexico border region is characterized by dynamic population movements and socio-economic disparities facilitating TB transmission. Tijuana, Baja California, Mexico, a transit hub for northbound migrants and a city with high homelessness rates, provides a unique setting to assess LTBI treatment acceptability among mobile, underserved populations. Research is needed to inform effective public health interventions to mitigate LTBI and TB burdens in this region.

LTBI reactivation risk is elevated in key populations (e.g., persons with HIV, diabetes, and substance use disorders), and those who progress to active TB disease can infect others before being diagnosed and treated.^7^ Untreated LTBI affects individuals and public health, straining systems and facilitating TB spread. Preventive therapy significantly lowers progression risk by eliminating dormant *M. tuberculosis*. Recently approved regimens have shortened LTBI treatment from nine months to one, improving accessibility for hard-to-reach groups.^8^,^9^ Identifying LTBI-positive migrants and understanding their treatment willingness is crucial for TB prevention at the border and beyond.

This study assessed unwillingness to receive LTBI treatment if indicated among migrants in Tijuana and examined associated factors. We explored potential barriers and facilitators impacting an individual’s decision to initiate and adhere to treatment. Findings contribute to TB epidemiology knowledge in border regions and may inform strategies to enhance screening, testing, and treatment among mobile populations.

## Methods

### Design and Setting

Data for this analysis were derived from a study on COVID-19’s impact on migrants in Tijuana. The study methods were published previously.^10^ Using a cross-sectional, non-probability sampling approach, we recruited and surveyed migrants living in and out of shelters from November-December 2020 and February-April 2021, with a pause due to COVID-19 distancing measures.

Participants surveyed met the following eligibility criteria: (a) age ≥ 18 years; (b) place of birth in a Latin American or Caribbean country other than Mexico and lived in Tijuana for ≤ 5 years, or born in a Mexican state other than Baja California and lived in Tijuana for ≤ 1 year due to internal displacement or deportation from the U.S.; and (c) ability to complete the survey in Spanish, English, or French.

Interviews were conducted in migrant shelters and a storefront called “La Frontera”. The shelter sample included shelter residents and non-residents accessing services (e.g., food, legal aid). La Frontera serves as a hub for research and harm reduction services for people who use drugs, many of whom are migrants.^11^ This recruitment approach produced two groups of participants: (1) in-transit migrants (those not intending to stay in Tijuana but were stopped there because of U.S. COVID-19-related migration policies); and (2) migrants (mainly from Mexico and the U.S.) connected with La Frontera. Shelter participants were more often women, while La Frontera participants included more drug users and unsheltered individuals.

### Data Collection

Participants completed an interviewer-administered questionnaire that assessed sociodemographic, migration, housing, substance use and health-related factors. Interviews were conducted in the participant’s preferred language using laptops.

To assess whether participants would be willing to receive treatment for LTBI if they tested positive, they were asked the question, “If the test is positive and suggests that you have the tuberculosis bacteria in your body (even without symptoms), would you be willing to take a one-month treatment course?” (Yes/No). Interviewers provided standardized explanations of LTBI diagnosis and treatment to ensure understanding. Since testing had not yet occurred, responses were unbiased by results.

Potential predictor variables were considered a priori that could affect participant willingness to receive LTBI treatment or identify groups requiring additional interventions to increase treatment acceptance. Considering migration-related variables, we assessed country of birth (Mexico vs. other), city or country where participants lived before coming to Tijuana, and having a goal of migration to the U.S. within the next year (Yes/No). Participants were asked if they had started migration procedures while in Mexico (e.g., for regularization or refugee status). The survey also assessed self-reported health status, chronic disease diagnoses, and need for healthcare in the past three months. We assessed alcohol and substance use as follows. High-risk drinking was defined as having 5 or more drinks per occasion or having 3 or more drinks at least 4 or more times per week for men and having 3 or more drinks per occasion for individuals not identifying as men.^12^ We assessed lifetime and current (past 6 months) use of the most common drugs in Tijuana (i.e., heroin, crack/powder cocaine, and methamphetamine).^11^ Sociodemographic variables included gender, age, being a member of an indigenous group or Afro-descendant, education. Housing stability in the past three months considered those who lived only in a house, apartment, or rented room in a hotel, motel, or rooming house that was not a private home as having stable housing versus reporting any other living situation. Due to the small number of transgender participants, we could not analyze this group independently. Since willingness to receive LTBI treatment was considered more likely influenced by gender identity as a social construct rather than biological sex, we grouped 3 transgender female-to-male participants with males, and 7 transgender male-to-female participants together with 3 participants who reported their gender as ‘other’ in the non-male category for all analyses. This approach is consistent with practices in epidemiologic research on gender measurement.^13^,^14^ Since one objective of this study was to inform potential future interventions intended to increase LTBI treatment for this population, we asked participants who were willing to receive LTBI treatment about treatment importance, barriers, and facilitators. To minimize participant burden from lengthy interviews, these items were not asked of participants who reported being unwilling to receive treatment.

### Tuberculosis Testing

LTBI status was assessed using the Mantoux tuberculin skin testing (TST) following standard recommended procedures.^15^ Purified protein derivative (PPD) was placed subcutaneously on the participant’s forearm during the study interview visit. Participants were instructed to return within 48–72 h for evaluation. The reaction was assessed by measuring the diameter of induration in millimeters. An induration of ≥ 10 mm was considered positive except when the participant was known to be living with HIV, in which case ≥ 5 mm was considered positive. Those who tested positive were referred to Tijuana TB control program clinics for further evaluation to rule out active TB disease and offered LTBI treatment if eligible per Mexican guidelines.^16^ Trained professionals administered and read tests, and PPD tuberculin potency and storage were checked regularly per manufacturer’s recommendations.

### Statistical Analysis

The primary outcome measure was self-reported unwillingness to complete a one-month treatment course if treatment was indicated. We compared characteristics of willing vs. unwilling participants using Chi-square and Fisher’s exact tests for categorical variables and t-tests for continuous variables. Statistical significance was set at *p* < 0.05. Given the high prevalence of unwillingness, Poisson regression with robust variance estimation (GEE) was used to identify associated factors.^17^ All statistically significant univariate variables (*p* < 0.05) were considered for multivariable modeling, assessing collinearity and interactions. The final model included only significant covariates (*p* < 0.05). Analyses were conducted in STATA, version 17 (College Station, TX).

### Compliance with Ethical Standards

Participants provided written informed consent in their preferred language. Ethics approvals were obtained from El Colegio de la Frontera Norte (066–160720) and University of California San Diego (201153).

## Results

### Participant Characteristics

A total of 598 interviews were completed, which included 481 (80.4%) migrants living in shelters (resident), 21 (3.5%) migrants recruited at shelters but living independently (non-resident), and 96 (16.1%) migrants recruited through La Frontera. One shelter participant with prior LTBI treatment and two shelter participants who did not answer the treatment willingness question were excluded. Of the remaining 595 participants, 71 (11.9%) overall responded that they were unwilling to take a one-month treatment course for LTBI if they tested positive (Table 1), which was much higher among participants from La Frontera compared to shelters (64.6% vs. 1.8%, *p* < 0.001). Retention for TST reading was 89.9% overall and was higher for La Frontera (100%) compared to shelter (87%) participants. The prevalence of TST-positivity was 18.3% overall, which was higher among participants from La Frontera (44.8% vs. 13.2%, *p* < 0.001) and among those unwilling to receive treatment (44.7% vs. 14.9%, *p* < 0.001, data not shown in table).

The mean participant age overall was 34.4 years (standard deviation: 10.6). Half (51.3%) were male, 29.6% completed high school or equivalent, 20.8% identified as Indigenous, 11.3% identified as Black, and 5.4% were stably housed (Table 1). Overall, 62.5% of participants were born outside of Mexico and 22.9% arrived in Tijuana from the U.S. (groups not mutually exclusive). Nearly half (47.4%) reported having spent time in migration detention in the U.S., 25.6% had started migration procedures while in Mexico, and 76.1% planned to migrate to the U.S. within the next year. Two-thirds (66.7%) rated their health status as “good” or “very good”, with one-third (35.6%) requiring healthcare in the past three months and 18.0% reporting ever being diagnosed with a chronic disease. Substance use was low overall. 21% reported smoking cigarettes daily and 14.8% reported levels of alcohol use considered high risk. Marijuana was the most widely used illegal substance ever in participants’ lifetimes (20.8%) followed by heroin (16.8%), methamphetamine (13.6%), and cocaine (6.9%); less than 10% reported using any one of these substances in the past 6 months.

Compared to shelter participants (Table 1), La Frontera participants were more likely to be older, identify as male, have arrived in Tijuana from the U.S. (primarily deportation), detained in a U.S. immigration facility, unstably housed, current cigarette smokers, and drug users (methamphetamine, marijuana, or heroin). They were less likely to have completed high school, to be Indigenous or Black, have been born outside of Mexico, arrived in Tijuana from their place of birth, started migration procedures in Mexico, planned to migrate to the U.S., self-reported a chronic disease, or required healthcare in the past three months (all p-values < 0.001). Only self-reported health status, high-risk alcohol consumption, and crack/cocaine use were similar across groups.

To assess potential barriers and facilitators to LTBI treatment, we analyzed responses from all participants who were willing to receive treatment if TST-positive (*n* = 524). Nearly all (98.6%) shelter participants and 70.5% of La Frontera participants reported that treatment would be “important” or “very important” (Table 2). Barriers such as remembering to take pills, fear of authorities, and pill storage issues were more commonly reported by La Frontera participants. Only shelter participants reported concerns about being judged for infection, fear of side effects, and other reasons as barriers to treatment. Regarding interventions to support LTBI treatment completion, cash incentives were the most frequently cited facilitator by shelter participants (31.4%), whereas La Frontera participants most often cited case management (23.5%). Directly observed therapy was preferred by 21.8% of shelter participants, compared to only 5.9% of La Frontera participants.

Given the low prevalence of unwillingness for treatment among shelter participants (1.8%) and significant differences compared to La Frontera participants, further analysis of factors associated with treatment unwillingness was restricted to La Frontera participants (*n* = 96), of which 62 (64.6%) were unwilling to take treatment. We calculated unadjusted prevalence ratios (PR) and 95% confidence intervals (CI) using Poisson regression to identify factors associated with treatment unwillingness (Table 3). Bivariate analysis showed unwilling participants were more likely to be younger (PR = 0.98; CI: 0.96, 1.00 per year increase), have self-reported health status of “good” or “very good” (PR = 2.36; CI:1.40, 3.99), current high-risk alcohol use (PR = 1.49; CI: 1.15, 1.91), used heroin (PR = 1.58; CI: 1.26, 1.98), and methamphetamine use in the past six months (PR = 1.58; CI: 1.26,1.98). In multivariable analysis, only self-reported health status and heroin use in the past six months remained independently associated with unwillingness to receive treatment (*p* < 0.05).

## Discussion

Most migrants in shelters (98.2%) expressed willingness to take a one-month LTBI treatment course, while two-thirds (65.4%) of those recruited from La Frontera were unwilling. Notably, while migrants recruited at La Frontera were three times more likely to test TST-positive compared to migrants recruited from shelters, they were substantially less likely to be willing to accept treatment. Differences in treatment willingness between these groups may reflect differences in sociodemographic, migration, and behavioral factors.

Among migrants recruited from La Frontera, treatment unwillingness was associated with perceived good health status and recent heroin use. Knowledge of these factors can help target LTBI screening and treatment services for migrants. Perceiving treatment as “important” or “very important” was common among willing participants from both recruitment sites; however, differences in treatment barriers and facilitators suggest targeted interventions are needed to improve uptake and completion in specific migrant groups.

High prevalence of LTBI has been reported previously among people who inject drugs and unhoused individuals in Tijuana.^18^ Although the current study lacked data on TB disease prevalence among individuals who test TST-positive, the prior study found that only 2.8% of those who tested positive had TB symptoms, of which 14% tested sputum smear-positive; thus, we have high confidence that the majority of TST-positive participants in this study had LTBI.

Substance use is known to affect adherence to LTBI treatment.^19^,^20^ This study found that heroin use was a key factor associated with unwillingness to take treatment. One explanation is that chaotic living conditions among people in Tijuana who use heroin may make healthcare access difficult and LTBI treatment a low priority. Reported barriers to treatment included remembering to take medication, having no place to store medications, and fear of authorities. Patients with LTBI are advised to avoid alcohol during treatment due to hepatotoxicity risks, but high-risk alcohol use was not independently associated with unwillingness to take treatment. Many participants may have been unaware of this restriction when surveyed; however, in practice, some heavy alcohol users may decline treatment when advised to avoid alcohol. We also found that participants who smoked cigarettes daily were over three times more likely to be unwilling to receive LTBI treatment which aligns with previous studies linking smoking to lower adherence rates.^21^ Addressing substance use in interventions may help improve engagement and retention in LTBI care.

Individuals deported from the U.S. and interviewed at the storefront site—many of whom experience homelessness and substance use in Tijuana—face an elevated risk of LTBI reactivation, yet they were less willing to receive LTBI treatment compared with participants who had not been deported. Given the low incidence of TB in the U.S. and California.^22^, ^23^ some deported individuals may not perceive themselves as at risk, despite testing positive. Reducing TB transmission in Tijuana, the Mexican state with the highest TB incidence. 24 requires strategies to increase LTBI treatment accessibility, enhance perceived treatment importance, and minimize adherence barriers.

Several limitations must be considered. First, its cross-sectional design precludes inference about temporality or causality between exposures and willingness to initiate LTBI treatment. Findings represent a single point in time and may not reflect changes in attitudes or behaviors over time. Second, because participants were recruited using non-probability sampling methods, the sample may not be representative of the broader population. Consequently, selection bias is possible, and the generalizability of our findings to other groups should be interpreted with caution. However, the study was open to all migrants recruited from shelters throughout Tijuana, including those who were not shelter residents, supporting the presumption that the sample is broadly representative of unstably housed migrants. Third, participants who tested TST-positive were referred to a municipal health clinic for further TB evaluation and no treatment was provided by the study. Therefore, the findings on willingness to take treatment for LTBI may not accurately reflect treatment uptake if actually offered. Fourth was the small number of participants who reported a gender identity other than male or female, which required grouping decisions that may not fully capture variation across gender categories. Consequently, results pertaining to females should be interpreted with caution, as inclusion of a small number of ‘other’ responses within the non-male category could influence comparisons. Larger studies are needed to more precisely examine willingness to initiate treatment by gender identity. Finally, reasons for treatment unwillingness were not assessed, as only willing participants were asked about barriers and facilitators to treatment. Future qualitative studies should explore individual and contextual factors influencing treatment decisions may inform strategies to improve acceptance among migrants.

Despite these limitations, the study has several notable strengths. To our knowledge, it is among the few investigations to examine willingness to initiate LTBI treatment in this population, addressing an important gap in the literature. The use of standardized measures and structured data collection enhances the internal validity of the findings. These strengths support the relevance of our findings and provide a foundation for future studies using larger, probability-based samples and longitudinal designs.

Migrants are an important population for LTBI screening and treatment interventions. Most migrants in Tijuana were willing to receive LTBI treatment. Upon arrival in the U.S. through formal channels, they would be referred for treatment. For those who enter the U.S. informally, remain in Mexico, or return to their state or country of origin, short-course LTBI treatment could reduce TB progression and transmission risks, particularly in shelters, detention centers, and other congregate settings. TB prevention interventions that target migrants in the U.S./Mexico border region could have wide-reaching impact on reducing the spread of TB. The availability of a one-month LTBI treatment regimen increases the feasibility of completing treatment before migrants leave Tijuana. Tailored interventions, including incentives and adherence support, may improve uptake and completion rates.

## Conclusions

Migrants living in Tijuana shelters had a low prevalence of LTBI, and most were willing to be treated if diagnosed, suggesting TB screening and treatment among shelter residents could reduce reactivation risk and transmission. In contrast, La Frontera migrants, despite higher LTBI prevalence, were less willing to receive treatment. Addressing treatment barriers and facilitators, particularly among high-risk groups, is essential. Interventions should focus on increasing the perceived importance of treatment. This study underscores the need for targeted public health strategies to improve LTBI care for migrants in the U.S./Mexico border region, ultimately aiding TB control and prevention efforts in both countries.

### New Contribution to Literature

This study provides novel insights into willingness to receive latent tuberculosis infection (LTBI) treatment among internal and international migrants in Tijuana, Baja California, Mexico. While prior research has examined LTBI prevalence and treatment adherence among migrant populations, this study uniquely highlights disparities in treatment willingness in a critical transit region at the U.S./Mexico border. Our findings reveal that migrants residing in shelters overwhelmingly expressed willingness to receive short-course LTBI treatment (98.2%), whereas two-thirds (64.6%) of those recruited from a storefront providing harm reduction services for people who use drugs were unwilling to take treatment. Moreover, treatment unwillingness was significantly associated with heroin use in the past six months and self-perceived good health status. These findings expand the literature on TB prevention by demonstrating how social determinants, including homelessness and substance use, influence attitudes toward LTBI treatment in migrant populations.

### Implications for Practice and Policy

Given that migrants at the highest risk for TB reactivation were the least willing to receive LTBI treatment, tailored interventions are needed to improve treatment uptake in this population. Public health strategies should incorporate harm reduction approaches, including targeted outreach and case management, to engage migrants who use drugs in TB care. Additionally, addressing structural barriers such as unstable housing and limited healthcare access may improve treatment willingness. Policymakers should consider integrating LTBI treatment into broader migrant health services at the U.S./Mexico border to prevent TB transmission and reduce disease burden in both countries. Further research is needed to assess the feasibility and effectiveness of interventions that mitigate barriers to LTBI treatment among high-risk migrants.

## Figures and Tables

**Table 1:** Participant characteristics by recruitment venue (*n* = 595)

	Total *n* (%)	Shelter *n* = 499	Storefront *n* = 96	*p*-value
***Sociodemographics***				
Age (years), mean (SD)	34.4 (10.6)	33.5 (10.72)	39.2 (8.68)	< 0.001
Gender – Male^b^	305 (51.3)	221 (44.3)	84 (87.5)	< 0.001
Completed high school or equivalent	176 (29.6)	172 (34.5)	4 (4.2)	< 0.001
Indigenous	124 (20.8)	123 (24.7)	1 (1.0)	< 0.001
Black, Afro-descendant, or Garifuna	67 (11.3)	66 (13.2)	1 (1.0)	0.001
***Country of Origin and Migration***				
Born outside of Mexico	372 (62.5)	372 (74.6)	0 (0.0)	< 0.001
Arrived in Tijuana directly from place of birth	250 (42.0)	250 (50.1)	0 (0.0)	< 0.001
Arrived in Tijuana from the US	136 (22.9)	44 (8.8)	92 (95.8)	< 0.001
Time in US migration detention	282 (47.4)	201 (40.3)	81 (84.4)	< 0.001
Started the migration procedure in Mexico	152 (25.6)	152 (30.5)	0 (0.0)	< 0.001
Mid-term^c^ migration goal to enter the US	453 (76.1)	395 (79.2)	58 (60.4)	< 0.001
***Housing and Health Status***				
Stably housed in the past 3 months	32 (5.4)	20 (4.0)	12 (12.5)	0.001
Self-reported health status good/very good	397 (66.7)	331 (66.3)	66 (68.8)	0.645
Self-reported chronic disease diagnosis	107 (18.0)	101 (20.2)	6 (6.3)	0.001
Needed healthcare in past 3 months	212 (35.6)	210 (42.1)	2 (2.1)	< 0.001
***Substance Use***				
Current daily smoker	127 (21.3)	49 (9.8)	78 (81.3)	< 0.001
Current high-risk alcohol use	88 (14.8)	77 (15.4)	11 (11.5)	0.315
Marijuana use, ever	124 (20.8)	86 (17.2)	38 (39.6)	< 0.001
Heroin use, ever	100 (16.8)	13 (2.6)	87 (90.6)	< 0.001
Crack/cocaine use, ever	41 (6.9)	32 (6.4)	9 (9.4)	0.294
Methamphetamine use, ever	81 (13.6)	26 (5.2)	55 (57.3)	< 0.001
Marijuana use, past 6 months	56 (9.4)	28 (5.6)	28 (29.2)	< 0.001
Heroin use, past 6 months	20 (3.4)	5 (1.0)	15 (15.6)	< 0.001
Crack/cocaine use, past 6 months	5 (0.8)	4 (0.8)	1 (1.0)	0.813
Methamphetamine use, past 6 months	25 (4.2)	11 (2.2)	14 (14.6)	< 0.001
***Tuberculosis***				
Unwilling to be treated for LTBI^d^	71 (11.9)	9 (1.8)	62 (64.6)	< 0.001
Result of TST				< 0.001
Positive	109 (18.3)	66 (13.2)	43 (44.8)	
Negative	421 (70.8)	368 (73.8)	53 (55.2)	
Not read	65 (10.9)	65 (13.0)	0 (0.0)	

US, United States; LTBI, latent tuberculosis infection; TST, tuberculin skin test

a n (%) represent the affirmative response to the interview question

b Includes 3 transgender female-to-male participants; 7 participants reporting transgender male-to-female and 3 reporting “other” were coded as non-male

c Mid-term refers to in the next few months to one year from the time of the interview

d Willing to be treated for LTBI was assessed prior to testing for LTBI

**Table 2:** Potential barriers to initiating, and facilitators for completing, a one-month course of treatment for LTBI stratified by recruitment site among participants who said they would be willing to receive treatment if indicated

If you tested positive for having infection with the TB germs, but not TB disease, how important would it be for you to get treatment to kill the TB germs?	Storefront (*N*=34) *n* (%)	Shelter (*N*=490) *n* (%)
Not at all important	0 (0.0)	1 (0.2)
Somewhat important	10 (29.4)	6 (1.2)
Important	16 (47.1)	91 (18.6)
Very Important	8 (23.5)	391 (80.0)
***Which do you think would prevent you from being willing or able to take a one-month treatment course to prevent active TB? (check all that apply)***	**n (%)**	**n (%)**
Remembering to take pills	18 (52.9)	83 (16.9)
Concerns about being judged for having an infection	0 (0.0)	80 (16.3)
Fear of side effects	0 (0.0)	77 (15.7)
Encounters with authorities	6 (17.7)	35 (7.1)
Having a place to store pills	7 (20.6)	27 (5.5)
Other reason	0 (0.0)	12 (2.5)
None	12 (35.3)	280 (57.1)
***If you were given a 1-month treatment course to kill the TB germs and prevent them from causing TB disease in the future, which of the following would help you to complete the treatment? (check all that apply)***	**n (%)**	**n (%)**
Cash incentives	3 (8.8)	154 (31.4)
Peer-based DOT	0 (0.0)	107 (21.8)
Case management	8 (23.5)	96 (19.6)
Clinic-based directly observed therapy (DOT)	2 (5.9)	60 (12.2)
Video DOT	0 (0.0)	43 (8.8)
None of the above	22 (64.7)	151 (30.8)

**Table 3:** Bivariate and multivariable analysis of participant characteristics by unwillingness to be treated for LTBI among individuals recruited from the La Frontera storefront (*n*=96)

	Not willing to be treated (*n*=62)	Willing to be treated (*n*=34)	Bivariate Cluster Adjusted PR (95%CI)	*p*-value	Adjusted final PR (95% CI)
Sociodemographic Factors					
Age (years), mean *(SD)*	37.8 (8.8)	41.9 (8.0)	0.98 (0.96, 1.00)	**0.011**	
Gender – Male^a^	56 (90.3)	28 (82.4)	0.75 (0.42, 1.35)	0.338	
Completed high school or equivalent	2 (5.9)	2 (3.2)	0.77 (0.28, 2.08)	0.601	
Indigenous	0 (0.0)	1 (2.9)	-	-	
Black, afro descendant or Garifuna	1 (16)	0 (0.0)	-	-	
***Country of Origin and Migration***					
Born outside of Mexico	0 (0.0)	0 (0.0)	-	-	
Arrived in Tijuana directly from place of birth	0 (0.0)	0 (0.0)	-	-	
Arrived in Tijuana from the US	62 (100.0)	30 (88.2)	-	-	
Served migration detention in the US	51 (82.3)	30 (88.2)	0.86 (0.61, 1.22)	0.393	
Started the migration procedure in Mexico	0 (0.0)	0 (0.0)	-	-	
Mid-term^b^ migration goal to enter the US	37 (59.7)	21 (61.8)	0.97 (0.72, 1.31)	0.841	
***Housing and Health Status***					
Stably housed in the past 3 months	5 (14.7)	7 (11.3)	0.89 (0.54, 1.48)	0.654	
Self-reported health status good/very good	52 (83.9)	14 (41.2)	2.36 (1.40, 3.99)	**0.001**	2.37 (1.52, 3.99)
Self-reported chronic disease diagnosis	3 (4.8)	3 (8.8)	0.76 (0.34, 1.73)	0.516	
Needed healthcare in past 3 months	0 (0.0)	2 (5.9)	-	-	
***Substance Use Factors***					
Current daily smoker	53 (85.5)	25 (73.5)	1.36 (0.83, 2.22)	0.219	
Current high-risk alcohol use	10 (16.1)	1 (2.9)	1.49 (1.15, 1.91)	**0.002**	
Marijuana use, past 6 months	19 (30.7)	9 (26.5)	1.07 (0.78, 1.47)	0.660	
Heroin use, past 6 months	14 (22.6)	1 (2.9)	1.58 (1.26, 1.98)	**0.000**	1.70 (1.33, 2.19)
Methamphetamine use, past 6 months	6 (9.7)	8 (23.5)	0.63 (0.34, 1.17)	0.145	

a including transgender male-to-female

## Data Availability

The data from this manuscript are available upon reasonable request.
